# Outbreak investigation including molecular characterization of community associated methicillin-resistant *Staphylococcus aureus* in a primary and secondary school in Eastern Switzerland

**DOI:** 10.1038/s41598-022-24363-7

**Published:** 2022-11-18

**Authors:** Frederike Waldeck, Salome N. Seiffert, Susanne Manser, Danuta Zemp, Angela Walt, Christoph Berger, Werner C. Albrich, Matthias Schlegel, Tim Roloff, Adrian Egli, Oliver Nolte, Christian R. Kahlert

**Affiliations:** 1 Division of Infectious Diseases and Hospital Epidemiology, Cantonal Hospital St. Gallen, St. Gallen, Switzerland; 2 Division of Infectious Diseases & Microbiology, University Hospital Schleswig-Holstein, Campus Luebeck, Luebeck, Germany; 3 Division of Human Microbiology, Centre for Laboratory Medicine, St. Gallen, Switzerland; 4 Division of Public Health, Department of Health, Office of the Chief Medical Officer of Canton St. Gallen, St. Gallen, Switzerland; 5 Division of Public Health, Department of Health, Office of School Medicine, St. Gallen, Switzerland; 6 Division of Infectious Diseases and Hospital Epidemiology, University Children’s Hospital Zurich, Zurich, Switzerland; 7 Institute for Medical Microbiology, University of Zurich, Zurich, Switzerland; 8 Clinical Bacteriology and Mycology, University Hospital Basel, Basel, Switzerland; 9 Applied Microbiology Research, Department of Biomedicine, University of Basel, Basel, Switzerland; 10 Infectious Diseases and Hospital Epidemiology, Children’s Hospital of Eastern Switzerland, St. Gallen, Switzerland

**Keywords:** Bacterial infection, Risk factors

## Abstract

At our tertiary children’s hospital, infections with newly detected methicillin-resistant *Staphylococcus aureus* (MRSA) among children attending primary (age 6–12 years) and secondary school (age 13–16 years) nearly doubled in 2018 compared to previous years. This observation initiated an epidemiological outbreak investigation including phenotypic (susceptibility testing) and genotypic (whole genome sequencing) characterization of the isolates. In addition, a cross-sectional study was conducted to determine source of the outbreak, colonization frequency and to identify risk factors for transmission using a questionnaire. As a result, 49 individuals were detected with 57 corresponding isolates. Based on the case definition combined with whole genome sequencing, a core cluster was identified that shared common genetic features and a similar antimicrobial susceptibility pattern (efflux-mediated macrolide resistance, tetracycline susceptibility along with presence of Panton-Valentine leukocidin). Epidemiologic evaluation identified a distinct school as a common risk factor. However, the source of the clustered infections within that school could not be further specified. No further cases could be detected after decolonization of infected and colonized children.

## Introduction

Over the last 25 years, infections with community associated methicillin-resistant *Staphylococcus aureus* (CA-MRSA) have increased all over the world. In Switzerland, MRSA detection rates decreased in recent years in adults, but increased in the younger population based on data from the Swiss antimicrobial resistance surveillance network (ANRESIS, http://www.anresis.ch)^1^. However, MRSA frequency in children admitted to hospitals is still low^2,3^ and general screening is not recommended. Most children colonized with CA-MRSA remain asymptomatic. Symptomatic children mainly present with skin and soft tissue infections (SSTI) infections^4^ and severe invasive infections are rarely seen. CA-MRSA infections are epidemic in some countries which may suggest, that CA-MRSA are more virulent and transmissible than hospital-associated MRSA strains^5^. CA-MRSA frequently carry the cytotoxic toxin Panton-Valentin Leukocidin (PVL) as a virulence factor associated with more severe clinical manifestations, although conflicting data exist on the role of PVL in the pathogenesis of CA-MRSA infections^6^. Clusters of sequence type 5 (ST5) PVL producing CA-MRSA strains in inpatient neonates^7^, in school-children^8^ and of different CA-MRSA strains have been previously described in Switzerland^9,10^.

For many years, pulsed-field gel electrophoresis (PFGE), *spa* typing and SCC*mec* typing alongside with multi-locus sequence typing (MLST) represented the primary MRSA typing techniques^11^. Nowadays, because of next-generation sequencing (NGS) techniques, healthcare facilities are capable of tracing outbreaks with higher resolution than ever before. Whole-genome sequencing (WGS) as part of a surveillance system can be used to monitor pathogens of concern within a healthcare facility, a region or even countrywide. Furthermore, the use of core genome (cg) MLST allows a more in-depth view inside the evolution and spread of MRSA.

A cluster of CA-MRSA infections was detected in an 80-bed tertiary children’s hospital in Switzerland with the number of CA-MRSA cases doubling compared to previous years in the first half of 2018. Furthermore, susceptibility testing of most of the CA-MRSA cases revealed an efflux-mediated, non-inducible macrolide-streptogramin B antibiotic resistance pattern and positivity for PVL, further supporting our observation of a possible cluster. In consequence, epidemiological and genomic outbreak investigations were initiated with the aims of identifying the source and risk factors for the transmission of CA-MRSA, to implement measures to prevent transmissions and to end the outbreak.

## Methods

### Outbreak investigation

Outbreak investigation included (i) microbiological case definition by microbiological culture with susceptibility testing to detect CA-MRSA, followed by WGS and (ii) detection of additional cases by cross-sectional study. This allowed for epidemiological investigation with evaluation of risk factors by questionnaires, case and outbreak description, and finally implementation of measures i.e., decolonization of CA-MRSA cases.

### Microbial culture and MRSA detection

Copan eSwabs™ (Copan, Brescia, Italy) were used for swabbing and sent to the laboratory immediately after sampling. Ten microliters of the 1 ml liquid amies medium included in a Copan eSwab™ and containing the swab sample were transferred into enrichment broth (Brain heart infusion (BHI) with 6% NaCl, manufactured in house) and incubated overnight. Of the enriched broth 10 μl was inoculated onto a chromID chromogenic MRSA plate (bioMérieux, Marcy l’Etoile, France) with a WASP instrument (Copan, Brescia, Italy). After incubating the plates for 24 h in the smart incubators of a WASPLab™ high-resolution images of media plates were inspected using the WASPLab™ WebApp software. Colonies, indicative for MRSA were identified by a software algorithm (“segregation”) and sent to reading and picking for further workup^12^. Identification was done with a MALDI-ToF instrument using the BDAL 9.0 database (MALDI Biotyper Smart System, Bruker Daltonics, Bremen, Germany) with the colony transfer method (“direct smear”). Colonies identified as *S. aureus* were further tested with a PBP2a (penicillin-binding protein 2a, which confers methicillin-resistance) lateral flow immunochromatography assay (Alere Clearview PBP2a Culture Colony Test, Alere Inc. Waltham MA, USA), which confirmed MRSA. A BD™ Phoenix instrument (Becton Dickinson, Sparks, MD, USA) with PMIC-88 cartridge (tailored for the susceptibility testing of staphylococci and other Gram-positive bacteria) was used for susceptibility testing, except vancomycin, which was tested with epsilometer (E-) test (bioMérieux, Marcy l’Etoile, France). Antimicrobial susceptibility testing (AST) data were interpreted according to respective EUCAST guidelines (version 8.0 or 9.0 in 2018 and 2019, AST data not shown). This procedure applies to all isolates processed by the Centre for Laboratory Medicine in St. Gallen. Five isolates were tested elsewhere (i.e. available as laboratory report, only) and the data could not be verified.

### Genomic evaluation by whole genome sequencing (WGS)

DNA extraction was performed using the QIAsymphony DSP DNA Mini Kit (QIAGEN GmbH, Hilden, Germany). DNA was quantified using the Qubit dsDNA BR HS Assay Kit and Qubit fluorometers (Invitrogen, https://www.thermofisher.com). WGS was performed using Illumina MiSeq with the Nextera XTlibrary preparation kit (Illumina Inc., USA), according to the manufacturer’s procedure (paired-end sequencing). After mapping the raw data against the reference genome, we retained reads with at least 90% sequence identity, from areas with an average coverage (unassembled) of more than 57 reads, and reads longer than 123 base pairs. Trimming and assembly of raw reads was performed using the Velvet assembler of the SeqSphere software (Ridom, https://www.ridom.de, version 7.7.5 using *S. aureus* cgMLST v1.3 and *S. aureus* v1.1). The analysis included MLST, cgMLST (1861 targets), *spa* typing, clonal complex typing and detecting resistance/virulence genes (*mecA*, macrolide resistance genes (*ermA, ermC, mphC, msrA*) and PVL (LukS-PV and LukF-PV)) by using SeqSphere. Coverage was at least 50-fold. We defined core clusters as groups of isolates with ≤ 5 different SNPs between neighboring isolates based on the cgMLST. To generate phylogenetic SNP trees, we used SeqSphere (Ridom; Münster, Germany) in the pairwise ignore missing values mode and an unweighted pair group method. The WGS data has been submitted to NCBI under the following submission number PRJNA738326.

### Case definition based on microbiological results

**Figure 1:**
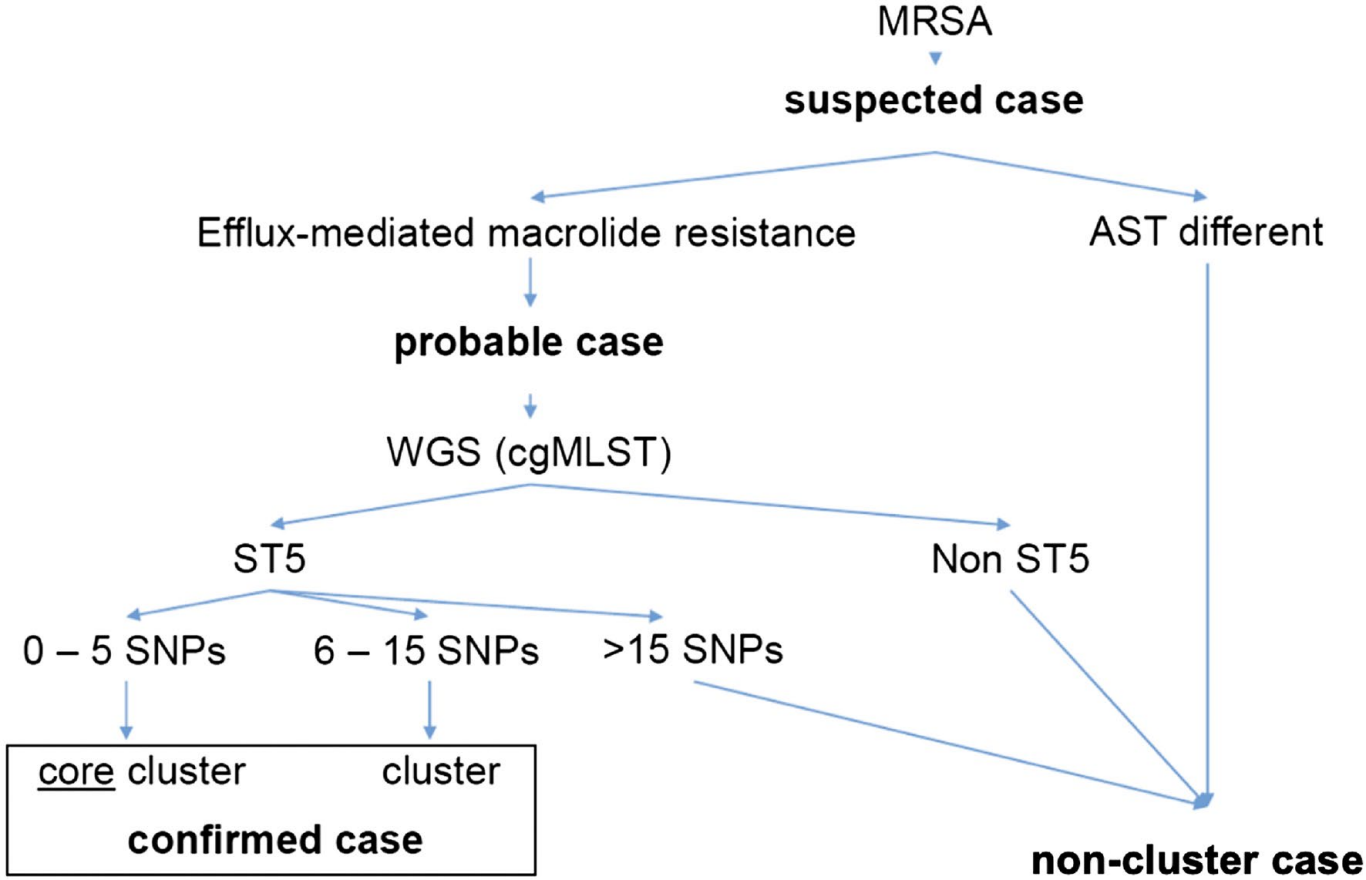
Case definitions based on microbiological results used in this study. *AST* antimicrobial susceptibility testing, *cgMLST* core genome multi-locus sequence typing, *WGS* whole-genome sequencing, *MRSA* methicillin-resistant *Staphylococcus aureus*, *MSSA* methicillin-susceptible *Staphylococcus aureus*, *ST* sequence type, *SNP* single nucleotide polymorphism.

The following case definition was used to categorize MRSA cases. First, all culture positive MRSA cases were “suspected cases”. Second, presence of the specific efflux-mediated macrolide resistance (phenotype) was assessed. Cases with an alternative antimicrobial susceptibility testing result were categorized as “non-cluster cases”. “Suspected cases” having efflux-mediated macrolide resistance were “probable cases”. In “probable cases” where strains were available, WGS was performed. As there are no international standards defining the cut-off between closely related and unrelated MRSA strains, we used the following two thresholds to categorize our strains^13^. If strains were ST5 using cgMLST, cases were “confirmed cases” and considered either as “core cluster” (≤ 5 single nucleotide polymorphisms (SNPs)) or “cluster” (> 5 but < 15 SNPs). All cases not belonging to ST5 or ST5 with > 15 SNPs were again classified as “non-cluster cases” (see Fig. 1).

### Identification of risk factors by questionnaires

Potential risk factors for CA-MRSA acquisition were assessed by two questionnaires before (column “Questionnaire” and during (column “Modified questionnaire”) the cross-sectional study and are provided in the Supplementary Table S1. The first questionnaire (“Questionnaire” was sent in spring to summer 2019 by email and/or mail to the families of confirmed and probable cases. Questions covered schooling (nursery, primary and secondary school), contact with the health-care systems (e.g., inpatient care), current, previous, or recurrent infections with MRSA or skin infections of the case and household contacts, profession, workplace, pets, leisure activities including sports, events, school or holiday camps or trips and attendance at the local children’s festival held in June 2018. The second questionnaire (“Modified questionnaire”) was used during the cross-sectional study (see “Cross-sectional study” section) and was electronically filled-in on site.

### Cross-sectional study

Given persistent transmission throughout 18 months (2018–2019), a cross-sectional study was initiated to identify additional cases. Results of the epidemiological investigation and the first questionnaire supported the hypothesis that the outbreak was linked to a particular primary and secondary school. Accordingly, all students and all staff at the affected school with proven direct contact to the students (i.e. teachers, pre-schooling teachers) were included in the study in November 2019. Clinical examination for skin lesions was performed in all students and staff. Swabs for MRSA (from axilla/inguinal region, nose/throat, wounds) were taken from all individuals with SSTI on clinical examination. These individuals also had to answer a second questionnaire, which was adapted from the previous one in summer 2018. Questions focused on school activities and leisure activities. Furthermore, every fifth student and staff without symptoms of CA-MRSA infection were randomly assigned to the cross-sectional study to receive swabbing and answer the questionnaire.

### Decolonization of CA-MRSA positive individuals

Protocols for decolonization can lead to the reduction of colonization and CA-MRSA infections^14^. To decolonize skin and mucous membranes, disinfectant soaps (i.e. octenidine-containing wash lotion), nasal ointment (i.e. containing mupirocin) and oral gel (i.e. chlorhexidine gluconate) were applied to the skin, hair, nose and throat 1–2 times daily for five days. Furthermore clothes, bed sheets and towels had to be changed daily and washed at 40–60 °C in a washing machine.

### Statistics

Continuous variables were assessed by t tests or Mann–Whitney U tests as appropriate. For categorical values, comparison was done by Fisher’s exact test. Missing data were not imputed. A two-sided p value < 0.05 was considered as statistically significant. Given the small number of each outcome, only the univariate but not the multivariable analyses are reported. Data analysis was performed with SAS (version 9.3, SAS Institute Inc., Cary, NC, USA).

### Ethics approval and consent to participate

The study was conducted according to the guidelines of the Declaration of Helsinki, mandated, and supported by the responsible cantonal authorities, i.e. the Division of Public Health, Department of Health, St. Gallen, the highest medical supervisory authority in the canton. In addition, formal informed consent was waived because this study involved anonymized health-related data and sequencing data of bacterial strains both of which were included in the general consent that all “cases” gave upon admission to the hospital. Therefore, additional approval by the local ethical committee was not applicable.

## Results

### Outbreak description and epidemiological investigation

First cases of CA-MRSA were initially identified and treated at the local tertiary care children’s hospital. A common denominator was quickly identified during clinical examination and case history taking: most cases were students attending a school within the same city. As a result of case accumulation within one school, the school medical service and the responsible health department were involved, and hygiene measures (intensified hand hygiene and disinfection of surfaces) initiated at the affected school in October 2018. To increase awareness of further CA-MRSA cases, pediatricians and family physicians in the city area were informed of an CA-MRSA outbreak and encouraged to take skin swabs if a typical clinical skin infections were observed and to decolonize in case CA-MRSA was detected and forward results to study team. Furthermore, parents of children from this school received an information letter in spring 2019 on CA-MRSA and the request to visit their family doctor if the child or themselves were symptomatic. Also, a media release was published in the regional newspaper. Physicians and parents were notified once more when the cross-sectional study took place (“awareness campaign”).

**Figure 2:**
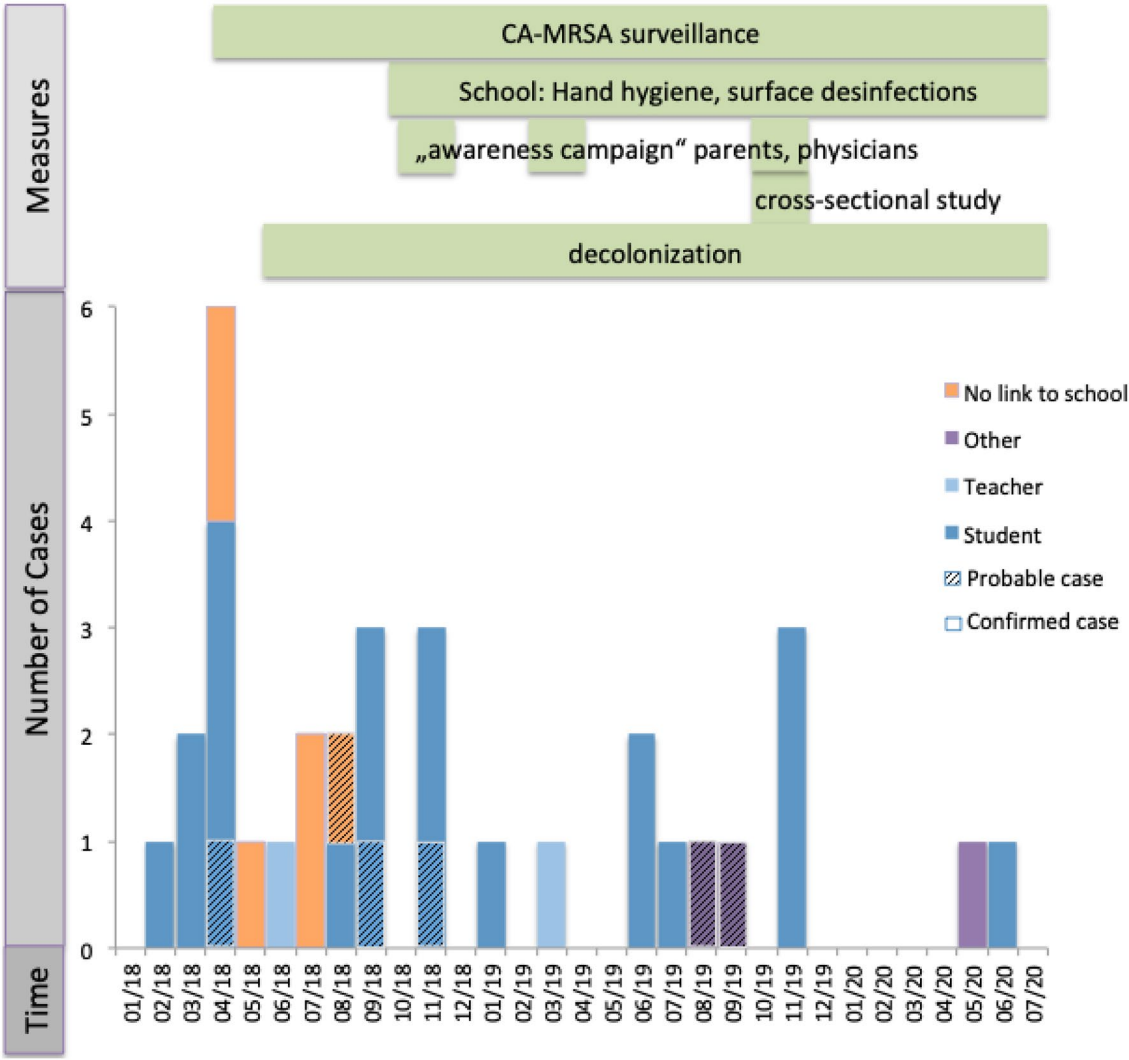
Epidemic curve of confirmed and probable cases with measures initiated during outbreak investigation (n = 33).

Although the number of cases decreased during the winter 2018/2019, new cases continued to occur (see Fig. 2). Hence, a first questionnaire was developed to identify other risk factors and determine venues in common and activities of cases. The questionnaire was distributed among 97% of confirmed and probable cases. Response rate was 65%, confirming that 23 (77%) cases were related to the school. There was no clustering regarding other risk factors of CA-MRSA than attendance of this school (see Supplementary Table S1). Affected children were not confined to a particular class, nor to any other shared venue or activity. As no additional risk factors were identified beyond attendance at this school, but additional infections were observed during the summer of 2019, a cross-sectional study was initiated (see below).

### Clinical description of cases

CA-MRSA was identified in 49 individuals from January 2018 until June 2020. Eight cases had two isolates (total 57 different isolates). Two children were hospitalized. One adolescent was hospitalized because of osteomyelitis and in addition to intravenous antibiotic administration, surgical procedures and drainage were required. Based on the case definition, 27 cases were classified as confirmed (ST5 according to WGS), 6 as probable (not available for WGS) and 16 were non-cluster cases (9 cases were non-ST5 and 7 had a divergent antimicrobial susceptibility pattern). The non-cluster isolates either showed a MLS_B_ phenotype with resistance to clindamycin (i.e., constitutive, or inducible), did not have macrolide resistance or had non-ST5 on whole genome sequencing.

Most cases of the outbreak were children and had mild clinical symptoms of MRSA infection. Clinical characteristics of the 33 probable and confirmed cases are provided in Table 1. Association with the affected school was observed in 79% of cases (see Fig. 1).

**Table 1:** Clinical characteristics of the CA-MRSA outbreak. Adults included teachers and parents and a teacher’s wife.

Characteristics	Children (n = 28)	Adults (n = 5)	All (n = 33)
Mean age [IQR], years	10.5 [9–12]	37 [33–48]	11 [9–14]
Female sex (%)	13 (46)	2 (40)	15 (45)
**Clinical diagnosis**			
SSTI	22	5	27
Bacteremia and arthritis	1	0	1
Bursitis	1	0	1
Other	1	0	1
Colonization*	3	0	3
Association with school (%)	21 (75)	5 (100)	26 (79)
Confirmed cases, ST5 (%)	24 (86)	3 (60)	27 (82)

*IQR* interquartile range, *SSTI* skin and soft tissue infection, *ST5* sequence type 5.

*Colonized students were identified in the cross-sectional study.

### Cross-sectional study

The cross-sectional study was performed between November 27–29, 2019, and 129 participants from the affected school were screened (104 students and 25 staff including teachers). SSTI were observed in 17 students (13%) but in no staff. MRSA infection was detected in 1.6% (2 students and no staff [p = 0.64]) and MRSA colonisation in 4.7% (6 students and no staff [p = 0.26]). Three of these six students had been symptomatic and identified as part of the MRSA cluster before but were not symptomatic at the time of the cross-sectional study. Skin infection was present both in participants with positive and negative MRSA results (p = 0.35). WGS revealed ST5 in all but one case from the cross-sectional study (87.5%). There was one non-cluster case (ST72). Using the modified questionnaire, we were again unable to identify risk factors for CA-MRSA colonisation (Supplementary Table S1) on univariate analysis comparing those with MRSA-detection to those without. All CA-MRSA positive individuals identified in the cross-sectional study (colonized with or without infection) underwent skin decolonization following local recommendations. Ongoing surveillance did not reveal new cases of CA-MRSA. Three individuals already known to have been colonized with CA-MRSA were found persistently colonized with CA-MRSA (Fig. 2).

### Results of WGS

**Figure 3:**
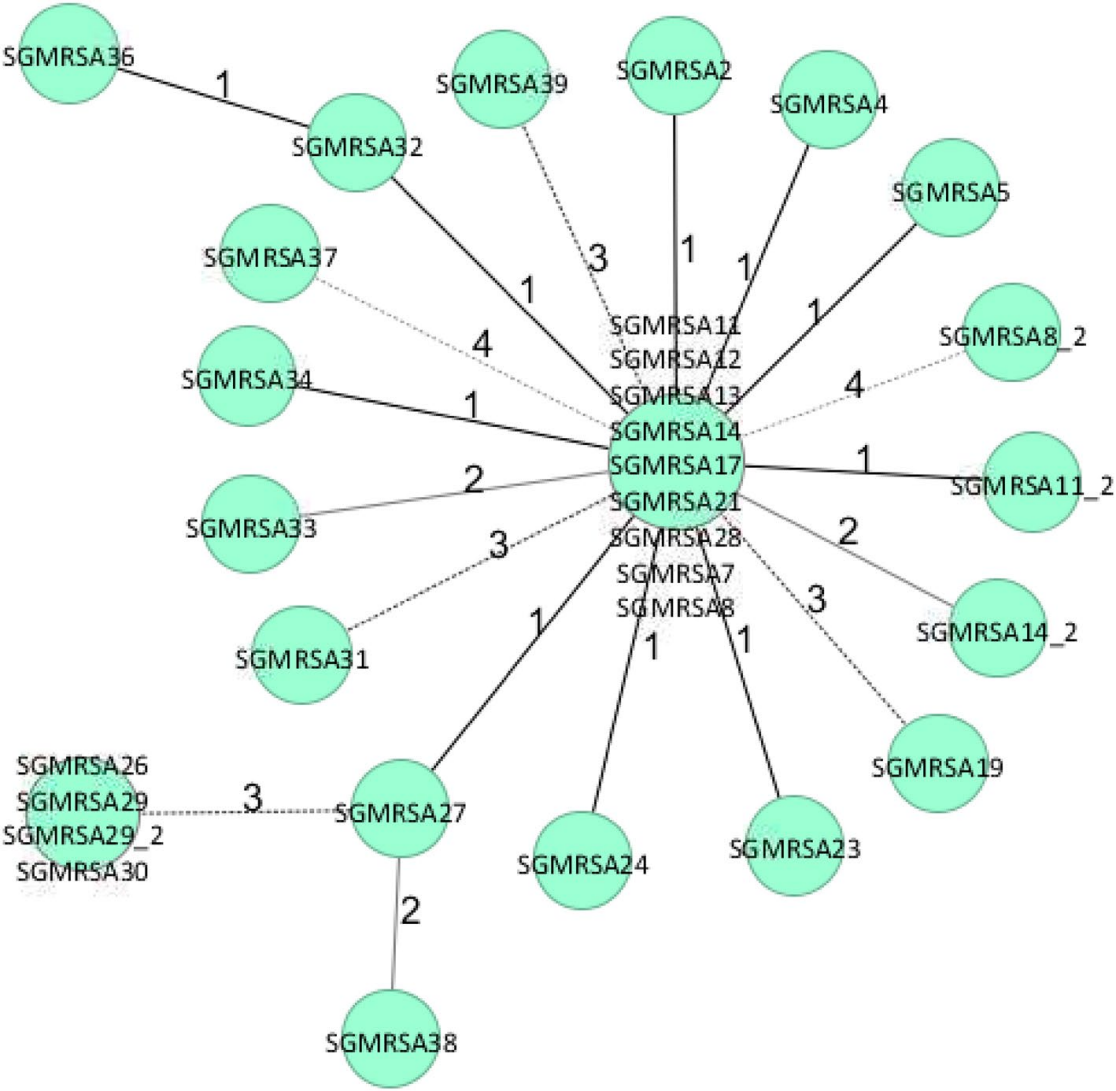
Phylogenetic single nucleotide polymorphisms (SNP) tree of the core cluster (N = 31).

**Figure 4:**
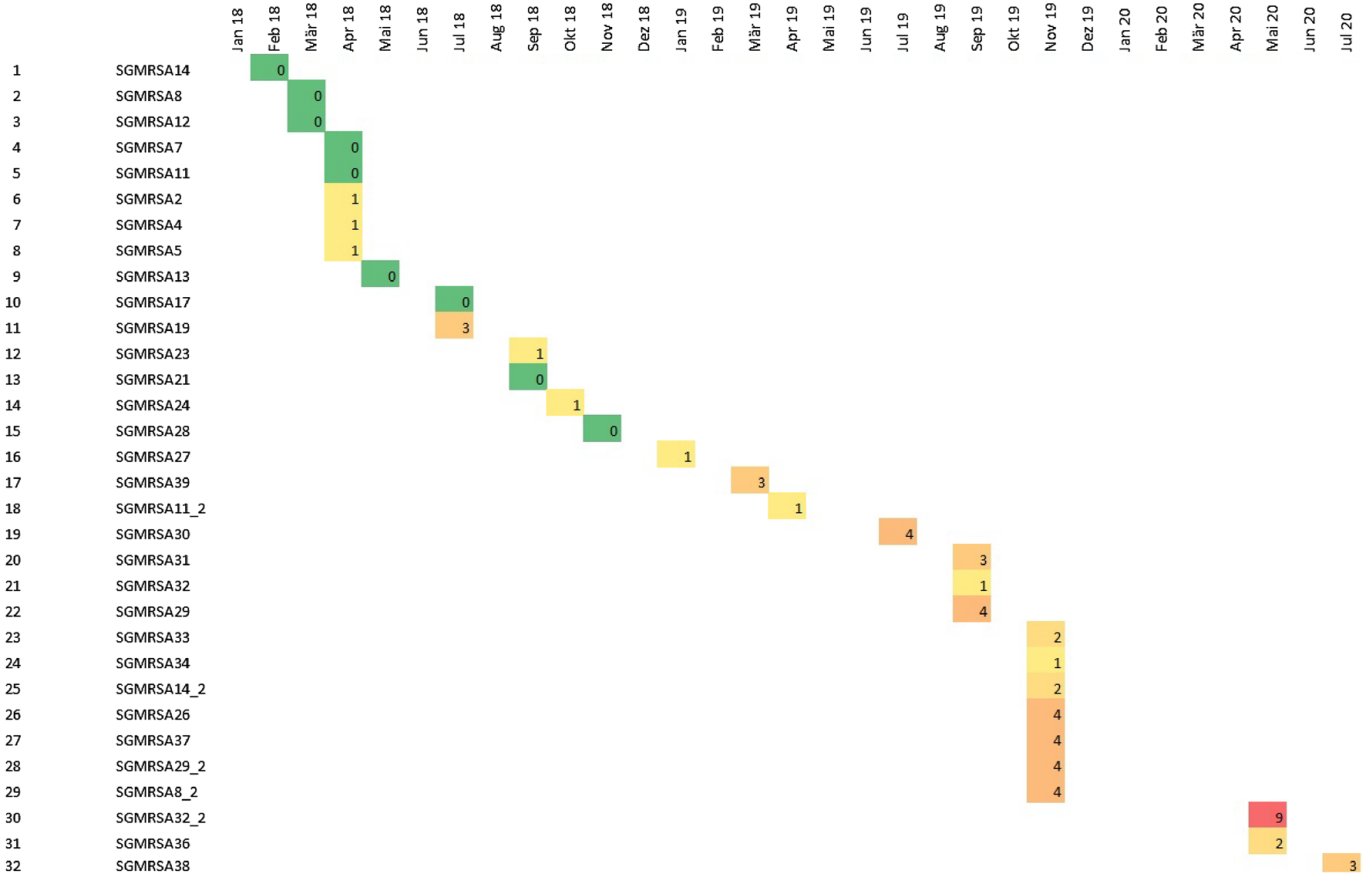
Graphical representation of micro-evolution within the ST5 cluster over time.

As a result of the outbreak investigation and the cross-sectional study, 39/49 cases (79%) were available for WGS (including seven individuals with two isolates) and 27/49 individuals (55%) belonged to the core cluster (31 isolates whereof 4 had an additional follow-up isolate). Results from WGS included MLST typing (sequence type), detection of mecA (Methicillin resistance gene), *spa* typing, clonal complex (CC) typing, the macrolide resistance genes (*ermA, ermC, mphC, msrA, vga, vgbB*), PVL genes (LukS-PV/LukF-PV) and the case definition (Table 2). 35 CA-MRSA isolates belonged to ST5 (*spa* type t002, CC5) and differed by a maximum of 259 SNPs. The outbreak cluster consisted of 31 isolates with a maximum of 9 SNPs (Fig. 3). The number of SNPs increased over time (Fig. 4). Within the sequenced ST5 isolates were five pairs from the same individual but 2 to 23 months apart, which also showed differences of 0,1, 2, 4, and 8 SNPs compared to the closest ST5 strain. This suggests evolution over time during this event. The methicillin and macrolide resistance genes (mecA, ermA, ermC, mphC, msrA) matched to their respective phenotypes. The macrolide resistance genes of the core cluster were a combination of the mphC and msrA genes, except for one isolate (SGMRSA37) that harbored no macrolide resistance genes and belonged to *spa* type t045. Besides the core cluster, several isolates harboring the MLS_B_ genes ermA and ermC were also discovered.

**Table 2:** Results of whole-genome sequencing of CA-MRSA isolates (N = 46).

Sample ID	Sampling date	ST	spa type	CC type	mecA	Macrolide resistance genes	PVL gene (Luk F/S)	Case definition	Source
SGMRSA1	02/2018	22	t3243	CC22	+	–	−	Non-cluster isolate	OI
SGMRSA1_2	02/2018	22	t3243	CC22	+	–	−	Non-cluster isolate	OI
SGMRSA2	04/2018	5	t002	CC5	+	mphC, msrA	+	Core cluster isolate	OI
SGMRSA3	02/2018	8	t008	CC8	+	–	+	Non-cluster isolate	OI
SGMRSA3_2	02/2018	8	t008	CC8	+	–	+	Non-cluster isolate	OI
SGMRSA4	04/2018	5	NA	CC5	+	mphC, msrA	+	Core cluster isolate	OI
SGMRSA5	04/2018	5	t002	CC5	+	mphC, msrA	+	Core cluster isolate	OI
SGMRSA6	04/2018	30	t632	CC30	+	ermA	−	Non-cluster isolate	OI
SGMRSA7	04/2018	5	t002	CC5	+	mphC, msrA	+	Core cluster isolate	OI
SGMRSA8	03/2018	5	t002	CC5	+	mphC, msrA	+	Core cluster isolate	OI
SGMRSA8_2	11/2019	5	t002	CC5	+	mphC, msrA	+	Core cluster isolate	CS
SGMRSA9	03/2018	22	NA	CC22	+	ermC	+	Non-cluster isolate	OI
SGMRSA10	02/2018	22	t910	CC22	+	–	−	Non-cluster isolate	OI
SGMRSA11	04/2018	5	t002	CC5	+	mphC, msrA	+	Core cluster isolate	OI
SGMRSA11_2	04/2019	5	t002	CC5	+	mphC, msrA	+	Core cluster isolate	OI
SGMRSA12	03/2018	5	t002	CC5	+	mphC, msrA	+	Core cluster isolate	OI
SGMRSA13	05/2018	5	t002	CC5	+	mphC, msrA	+	Core cluster isolate	OI
SGMRSA14	02/2018	5	t002	CC5	+	mphC, msrA	+	Core cluster isolate	OI
SGMRSA14_2	11/2019	5	t002	CC5	+	mphC, msrA	+	Core cluster isolate	CS
SGMRSA15	06/2018	5	t548	CC5	+	–	−	Non-cluster isolate	OI
SGMRSA16	06/2018	8	t008	CC8	+	–	−	Non-cluster isolate	OI
SGMRSA17	07/2018	5	t002	CC5	+	mphC, msrA	+	Core cluster isolate	OI
SGMRSA18	07/2018	5	t002	CC5	+	–	+	Non-cluster isolate	OI
SGMRSA19	07/2018	5	t002	CC5	+	mphC, msrA	+	Core cluster isolate	OI
SGMRSA20	11/2018	6584	t318	CC30	+	–	+	Non-cluster isolate	OI
SGMRSA21	09/2018	5	t002	CC5	+	mphC, msrA	+	Core cluster isolate	OI
SGMRSA22	09/2018	5	t002	CC5	+	ermA	−	Non-cluster isolate	OI
SGMRSA23	09/2018	5	t002	CC5	+	mphC, msrA	+	Core cluster isolate	OI
SGMRSA24	11-/2018	5	t002	CC5	+	mphC, msrA	+	Core cluster isolate	OI
SGMRSA25	08/2018	1472	t665	CC30	+	mphC, msrA	+	Non-cluster isolate	OI
SGMRSA26	06/2019	5	t002	CC5	+	mphC, msrA	+	Core cluster isolate	OI
SGMRSA27	01/2019	5	t002	CC5	+	mphC, msrA	+	Core cluster isolate	OI
SGMRSA28	11/2018	5	t002	CC5	+	mphC, msrA	+	Core cluster isolate	OI
SGMRSA29	06/2019	5	t002	CC5	+	mphC, msrA	+	Core cluster isolate	OI
SGMRSA29_2	11/2019	5	t002	CC5	+	mphC, msrA	+	Core cluster isolate	CS
SGMRSA30	07/2019	5	t002	CC5	+	mphC, msrA	+	Core cluster isolate	OI
SGMRSA31	08/2018	5	NA	CC5	+	mphC, msrA	+	Core cluster isolate	OI
SGMRSA32	06/2018	5	t002	CC5	+	mphC, msrA	+	Core cluster isolate	OI
SGMRSA32_2	05/2020	5	t002	CC5	+	mphC, msrA	+	Cluster isolate	OI
SGMRSA33	11/2019	5	t002	CC5	+	mphC, msrA	+	Core cluster isolate	CS
SGMRSA34	11/2019	5	t002	CC5	+	mphC, msrA	+	Core cluster isolate	CS
SGMRSA35	11/2019	72	NA	CC8	+	mphC, msrA	−	Non-cluster isolate	CS
SGMRSA36	05/2020	5	t002	CC5	+	mphC, msrA	+	Core cluster isolate	OI
SGMRSA37	11/2019	5	t045	CC5	+	–	+	Core cluster isolate	CS
SGMRSA38	07/2020	5	t002	CC5	+	mphC, msrA	+	Core cluster isolate	OI
SGMRSA39	03/2019	5	t002	CC5	+	mphC, msrA	+	Core cluster isolate	OI

+ presence of mecA, LukS/F; − absence of mecA, LukS/F; ST (Sequence Type) according to *S. aureus* multi-locus sequence typing (MLST); *spa* type (*S. aureus*-specific staphylococcal protein A) ; CC type (clonal complex); PVL (Panton-Valentine Leukocidin); NA (not available).

* Extension _2 means duplicate or follow-up isolate (depending on the date of isolation) of a given patient*

SNP tree generated by SeqSphere using the pairwise ignore missing values mode (46 samples with 28,657 columns) and an unweighted pair group method. Sample IDs refers to IDs provided in Table 2 and Fig. 4. Numbers close to connecting lines between the isolates represent the difference in the number of SNPs between isolates within the core cluster. The different connecting lines reflect the relatedness between the respective isolates. 27 confirmed cases plus 4 duplicates are shown; SGMRSA32_2 with 9 SNPs difference is not shown.

Isolates are stratified according to month of isolation. The number of SNPs observed with respect to the first isolate (SGMRSA14) is given. Note, that isolates obtained in November 2019 were from the cross-sectional study. Colors code the numbers of SNPs, with dark green for 0 SNPs. Increasing SNPs are colored from yellow to red.

## Discussion

This epidemiological and microbiological investigation of 49 children and adults with a newly detected CA-MRSA identified 33 cases that belonged to the outbreak. Molecular typing revealed a cluster with ST5 and a common efflux-mediated, non-inducible macrolide-lincosamide-streptogramin B (MLSB) antibiotic resistance pattern. Epidemiologic investigation found that in 79% of cases, a local elementary and secondary school (one campus) were the main sites of transmission. Following the implementation of interventions in the school and surrounding community as well as the conduct of a cross-sectional study with subsequent decolonization of participants in whom CA-MRSA could be found and no further cases occurred.

Although several CA-MRSA clusters have been described in Switzerland before, this is—to the best of our knowledge—the largest cluster of symptomatic children reported to date. The cluster also included 5 adults which had direct contact to the affected children and teachers (2 teachers, 1 father, 1 teacher’s wife, 1 mother). A cluster of ST5, SCC*mec* type IV PVL producing CA-MRSA strains has been previously described in Switzerland^7^. However, this cluster included seven inpatient neonates with a phenotypically different MRSA with respect to the antimicrobial resistance. In a surveillance report on 58 CA-MRSA cases from Western Switzerland, 13 distinct transmission clusters within 26 cases were detected over 3-year period^9^. About one fifth were children under 10 years of age and about two thirds of the isolates produced the PVL toxin. Finally a PVL-producing cluster of methicillin-susceptible *Staphylococcus aureus* within 10 of 22 schoolchildren and one of two teachers has been reported in Western Switzerland^8^. Six children were symptomatic with skin infections but none of the strains involved were MRSA. Similar to the cluster described here, no further cases occurred after implementation of treatment and decolonization. Further outbreaks with a ST5 PVL producing CA-MRSA strains have been described internationally^15^. To illustrate this, spread of a ST5^16^ has been linked to an increase of community onset staphylococcal diseases in children in Argentina and is a predominant clone in Australia^15^ and New Zealand^17^.

Molecular microbiological methods (i.e. WGS) enabled clear delimitation and description of this CA-MRSA ST5 cluster, providing important information for evaluating whether, where and for how long preventive measures are needed. This is highly useful information for managing an outbreak. Still, although we surveyed cases and families on risk factors by questionnaires (Supplementary Table S1) and conducted a cross-sectional study in the affected school, the key important insight on the common source of this CA-MRSA cluster remained unknown.

From the discussions during the investigations with the school representatives, the school medical service and the responsible health department, a hypothesis remains, which is supported by the WGS results. Initially, from February to November 2018, nine strains were identified by WGS with no difference in cgMLST (i.e. zero SNPs). These findings suggest an initial transmission event occurred in spring 2018.

We can only speculate about the exact timing and circumstances of this event. But it may have been in the context of a regional children’s festival in the spring of 2018, which takes place every 4 years. During rehearsals as part of the preparations, clothes worn directly on the skin were immediately changed between the children without being washed. Unfortunately, the questionnaire did not confirm this hypothesis since all children (with and without CA-MRSA) attended this event. However, given most children in the region participate, this is not unexpected. Further and less frequent transmissions during the following years must have taken part during school and leisure activities since CA-MRSA is mainly transmitted by direct contact.

Consistent with the epidemic curve (Fig. 2), the isolates had increasing numbers of SNPs over time compared to the first isolate in February 2018. While the actual magnitude of previous clusters reported from the literature remains unclear, we here provide evidence of a spatially and temporally restricted ST5 CA-MRSA PVL producing cluster.

Finally, we identified 4.7% CA-MRSA carriage on a school-based cross-sectional study during an outbreak and CA-MRSA infection in 1.6%. Data on the frequency of CA-MRSA carriage in the healthy population is not readily available since antimicrobial resistance testing according to the national antimicrobial resistance surveillance network ANRESIS is based on clinical isolates and not on swabs of healthy individuals. Thus, the rate of colonisation in healthy students with CA-MRSA in Switzerland is unknown. There are data from a Swiss single-center cross-sectional study in 340 hospitalized children with no MRSA detection^3^. Asymptomatic colonization was found in 0.1% of pre-clinical students in Warsaw, Poland which was distinctively lower than the CA-MRSA carriage rate in our population of students^18^. Higher rates have been observed in nursing homes (14.5–38%)^19,20^, intravenous drug users (8.7%)^21^ and in persons with contact with livestock (6.7%)^22^. In a worldwide study comprising of > 1000 surgeons only 2% were colonized with MRSA^23^ and 4.6% of health-care workers on meta-analysis^24^. We therefore consider the CA-MRSA carriage rate of 4.7% in healthy individuals to be above what would be expected in our setting. The identification of a single non-ST5 isolate on cross-sectional study (0.78%) that likely represents baseline carriage further strengthens our hypothesis. Overall, and although the ST5 strain produced PVL, the clinical phenotypes in the children were benign and non-invasive, except for one case with bacteremia and osteomyelitis.

Our study has several limitations: Since we have not systematically compared our data with a nationwide strain collection, we still have only a limited view of the “big picture”. However, we assume that for the majority of CA-MRSA-infections (i.e. SSTI) isolates are currently not characterized by cgMLST and nationwide analysis is likely to be biased towards isolates from the hospital setting. In addition, we did not conduct the cross-sectional study in a control school without a cluster of cases nor did we include a control group from the general population. Therefore, we are not able to compare the results with the MRSA carrier rate in asymptomatic school children. Moreover, the outbreak investigation was still taking a long time, so the actual additional benefit of rapidly available WGS results could not yet be demonstrated in this study. However, this may improve with faster availability of WGS in routine practice. Finally, while we could not determine the exact start of the cluster, transmission chains or independent predictors for the cluster. However, the observed microevolution (i.e. accumulation of SNPs) suggests an initial transmission event with accumulated SNPs over time, reflecting a molecular clock, driving genetic diversity.

There are several points that substantiate our findings. First, we did not only focus on the initial cluster but followed school classes longitudinally followed for over 2 years. Since single cases of CA-MRSA SSTI still occurred at that time, a rigid cross-sectional study was conducted. This allowed us not only to describe the cluster itself, but to analyze microevolution over time. Second, since the number of SNPs within the cluster was low as expected, we performed WGS of randomly chosen MRSA isolates over time to diversify the molecular data. This was done for three isolates, two of the cluster and one isolate of different ST and phenotype.

## Conclusion

We identified and characterized a school outbreak of ST5 PVL CA-MRSA in 33 individuals by epidemiological investigation combined with WGS. As detailed risk assessment and a cross-sectional case–control study revealed no other source of transmission than a local school, therefore transmission likely occurred during school activities. Our data suggest an initial transmission event that could have been the local children’s festival. After implementing hygienic measures, informing parents and local physicians and treating and decolonizing all infected and colonized CA-MRSA carriers, no further cases were detected during continued surveillance. A combined approach of epidemiological and microbiological methods is successful to terminate an outbreak of CA-MRSA.

## Supplementary Information

Supplementary Table S1.

## Data Availability

The datasets generated and/or analyzed during the current study are currently not publicly available. Deidentified participant data might be available on reasonable request by email to the corresponding author. The WGS data has been submitted to NCBI under the following submission number PRJNA738326.
