# Supplementary material for: Outbreak investigation including molecular characterization of community associated methicillin-resistant Staphylococcus aureus in a primary and secondary school in Eastern Switzerland
Source: Sci Rep. 2022 Nov 18;12:19826. doi: 10.1038/s41598-022-24363-7 (PMC9674615; doi:10.1038/s41598-022-24363-7)
Supplement: Supplementary file 1 — Supplementary Table S1. [file 41598_2022_24363_MOESM1_ESM.docx]

# Supplementary Material

Table S1: Possible risk factors for CA-MRSA acquisition assessed by questionnaires before (column “Questionnaire” and during (column “Modified questionnaire”) the cross-sectional study.

| Factors | Questionnaire | Modified questionnaire |
| --- | --- | --- |
| Personal data   - Age, sex - Postal code - Student or teacher? | ×  × | ×  ×  × |
| Household   - Number of household members including age - Known MRSA-infection or colonization of household contacts - Skin infection of household contacts in the recent year | ×  × | × |
| MRSA   - Known MRSA infection or colonization in the personal surroundings | × |  |
| Personal history   - Previous skin infections - Previous MRSA infections - Previous MRSA colonization - Treatment for MRSA or skin infections - Regular contact to the health system including hospitals, private practice, dialysis, psychiatric clinic, physiotherapist, nursing home - Persistent sign of infection (with MRSA) - Current skin infection | ×  ×  ×  ×  ×  ×  ×  × | ×  × |
| If the person is a child:   - contact to the Department of Neonatology, University Hospital Zurich, Switzerland - primary or secondary school (name, dates) - nursery, day care, kindergarden, play group - Job of parents / step parents - Job of student - Attendance of child care at MRSA effected school - Attendance of a children’s meeting at the affected school   If the person is an adult   - Job - partner’s job | ×  ×  ×  ×  ×  ×  × | ×  ×  × |
| Leisure and hobbies   - Sports: basketball, volleyball, soccer, swimming class, handball, soccer, hockey, martial arts, swinging*, athletics, fitness, fencing, gymnastics, riding, vaulting, other - Activities and events: children’s festival, school camp, ski camp, scouts’ trips, holiday camps, sport tournaments, work trips, swimming / whirl pool, others - Memberships or engagement in clubs or social events - Military or civil service - Other hobbies or leisure activities | ×  ×  ×  ×  × | ×*  ×  ×**  × |
| Animal contacts   - Contact to pets or other animals | × | × |

*soccer and dancing only, **membership in musical society
